# A comparative study on the effects of biodegradable high-purity magnesium screw and polymer screw for fixation in epiphyseal trabecular bone

**DOI:** 10.1093/rb/rbae095

**Published:** 2024-09-03

**Authors:** Liang Chang, Ying Luo, Weirong Li, Fangfei Liu, Jiaxin Guo, Bingyang Dai, Wenxue Tong, Ling Qin, Jiali Wang, Jiankun Xu

**Affiliations:** Musculoskeletal Research Laboratory, Department of Orthopaedics & Traumatology, The Chinese University of Hong Kong, Hong Kong, 999077, China; School of Biomedical Engineering, Sun Yat-Sen University, Guangzhou, Guangdong, 510000, China; Dongguan Eontec Co., Ltd, Dongguan, Guangdong, 510730, China; Dongguan Eontec Co., Ltd, Dongguan, Guangdong, 510730, China; Musculoskeletal Research Laboratory, Department of Orthopaedics & Traumatology, The Chinese University of Hong Kong, Hong Kong, 999077, China; Musculoskeletal Research Laboratory, Department of Orthopaedics & Traumatology, The Chinese University of Hong Kong, Hong Kong, 999077, China; Musculoskeletal Research Laboratory, Department of Orthopaedics & Traumatology, The Chinese University of Hong Kong, Hong Kong, 999077, China; Musculoskeletal Research Laboratory, Department of Orthopaedics & Traumatology, The Chinese University of Hong Kong, Hong Kong, 999077, China; School of Biomedical Engineering, Sun Yat-Sen University, Guangzhou, Guangdong, 510000, China; Musculoskeletal Research Laboratory, Department of Orthopaedics & Traumatology, The Chinese University of Hong Kong, Hong Kong, 999077, China

**Keywords:** high-purity magnesium screw, polylactic acid screw, peri-implant bone quality, epiphyseal trabecular bone, macrophage

## Abstract

With mechanical strength close to cortical bone, biodegradable and osteopromotive properties, magnesium (Mg)-based implants are promising biomaterials for orthopedic applications. However, during the degradation of such implants, there are still concerns on the potential adverse effects such as formation of cavities, osteolytic phenomena and chronic inflammation. Therefore, to transform Mg-based implants into clinical practice, the present study evaluated the local effects of high-purity Mg screws (HP-Mg, 99.99 wt%) by comparing with clinically approved polylactic acid (PLA) screws in epiphyseal trabecular bone of rabbits. After implantation of screws at the rabbit distal femur, bone microstructural, histomorphometric and biomechanical properties were measured at various time points (weeks 4, 8 and 16) using micro-CT, histology and histomorphometry, micro-indentation and scanning electron microscope. HP-Mg screws promoted peri-implant bone ingrowth with higher bone mass (BV/TV at week 4: 0.189 ± 0.022 in PLA group versus 0.313 ± 0.053 in Mg group), higher biomechanical properties (hardness at week 4: 35.045 ± 1.000 HV in PLA group versus 51.975 ± 2.565 HV in Mg group), more mature osteocyte LCN architecture, accelerated bone remodeling process and alleviated immunoreactive score (IRS of Ram11 at week 4: 5.8 ± 0.712 in PLA group versus 3.75 ± 0.866 in Mg group) as compared to PLA screws. Furthermore, we conducted finite element analysis to validate the superiority of HP-Mg screws as orthopedic implants by demonstrating reduced stress concentration and uniform stress distribution around the bone tunnel, which led to lower risks of trabecular microfractures. In conclusion, HP-Mg screws demonstrated greater osteogenic bioactivity and limited inflammatory response compared to PLA screws in the epiphyseal trabecular bone of rabbits. Our findings have paved a promising way for the clinical application of Mg-based implants.

## Graphical Abstract

**Figure:**
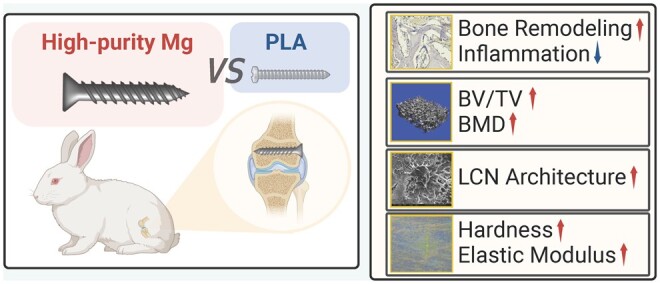

## Introduction

The global trend of increasing musculoskeletal disorders (MSDs), such as fracture, arthritis and ligament injury, brings formidable challenges to physical disability in our society [1–3]. In the USA, MSDs have become the leading cause of disability and accounted for the largest health expenditure since 2016, which was much greater than either cardiovascular diseases or cancers [4]. However, the limitations of traditional metallic implants/devices used in the orthopedic field may include their excessive stress shielding effects, implant loosening, peri-implant bone loss and demand for implant removal, substantially exacerbate patients’ physical, mental and financial burdens [5–10]. Thus, there is an urgent demand for research and development on developing innovative implants for orthopedic applications.

Based on the excellent biocompatibility, biodegradability and favorable mechanical and osteopromotive properties [11–16], magnesium (Mg)-based implants have drawn much attention to its potential clinical translation in the orthopedic field [14, 15, 17–21], especially for those challenging skeletal diseases associated with impaired bone healing, such as osteoporotic fractures, atypical femur fractures and periprosthetic fractures [19, 22–26]. Nonetheless, the insufficient mechanical strength and rapid degradation rate of Mg-based implants limit their applications, especially at high load-bearing skeletal sites. To overcome the above flaws, methods aiming at improving the corrosion resistance of Mg-based implants such as Mg purification have been explored [27, 28]. High-purity Mg (HP-Mg) (99.99 wt.%) materials offer comparable mechanical properties to Mg alloys without alloying toxic elements [29–33]. Furthermore, they exhibit enhanced corrosion resistance compared to most Mg alloys, which is attributed to lack of second phases and reduced micro-galvanic reactions, leading to improved mechanical support and reduced accumulation of hydrogen gas, particularly during the early-stage post-implantation [27–29].

To date, several studies have been conducted on HP-Mg screws, confirming their favorable long-term corrosion behavior and biocompatibility *in vivo* [29, 34, 35]. The Mg-based orthopedic screws have been recently successfully applied for hallux valgus surgery, fixation of the vascularized bone flaps, malleolar fractures and distal radius fractures in clinical trials in Germany, China and South Korea, respectively [36–40]. For animal experiments, the therapeutic effects of HP-Mg screws on the fractures of epiphyseal trabecular bone at load-bearing sites, such as femoral intracondylar fracture and femoral neck fracture, were also elucidated [29, 34]. Furthermore, Zhao *et al*. [38] validated the favorable outcome of treating avascular necrosis patients with vascularized bone grafting fixed by HP-Mg screw. However, since previous studies evaluated peri-implant bone quality mainly through histological and micro-CT analyses [29, 34, 35, 38], the deeper assessment about the effects of HP-Mg screw on the peri-implant trabecular bone tissue is still limited. For example, whether macrophage infiltration caused by phagocytosis of HP-Mg implant degradation products induces severe foreign body reactions (FBRs) at surface of implants is unknown [41–45]. Furthermore, the microstructure of peri-implant osteocytes, such as lacuno-canalicular network (LCN) architecture and canalicular junctions, could be observed to better evaluate cellular status [46–48].

In the present study, a comparative study was conducted to compare the effects of biodegradable HP-Mg screws (99.99 wt.%) and clinically used biodegradable polylactic acid (PLA) screws on peri-implant trabecular bone quality. PLA screw, a widely used biodegradable implant material, was employed as a reference to underscore the potential advantages of HP-Mg screw. Compared to polymer-based material, HP-Mg exhibits faster degradation rate and superior mechanical properties, including a higher tensile yield strength (160–240 MPa in Mg versus 50–100 MPa in PLA) and a greater Young’s modulus (41–45 GPa in Mg versus 3–4 GPa in PLA) [7]. Although Mg and PLA possessed distinct degradation process, both of them were reported to be associated with inflammatory reactions, FBRs and osteolytic phenomena [49–53]. In our study, we hypothesized that HP-Mg screw (Mg group) improved the quality of peri-implant bone tissues compared to PLA screw (PLA group). Specifically, screws were implanted at the femoral condyle of rabbits, the peri-implant bone remodeling dynamics, bone microstructure, biomechanical and histomophometric properties of mineral matrix were assessed at different time points. Besides, microscopic morphology of peri-implant osteocytes was also observed through scanning electron microscope (SEM) to assess their cellular status. In addition, finite element analysis (FEA) was applied to verify the potential of Mg orthopedic implants as the alternatives to their counterparts, such as PLA screws, through calculation of the stress distribution in the peri-implant bone tissue. Collectively, this study aimed to provide a comprehensive understanding of the beneficial effects of HP-Mg screws on the surrounding trabecular bone, thereby facilitating their clinical translation and expanding the range of available options for orthopedic surgeons in the treatment of epiphyseal bone defects or fractures.

## Materials and methods

### Fabrication of screws

The HP-Mg (99.99 wt.% Mg; 0.002 wt.% Al; 0.002 wt.% Mn; 0.003 wt.% Si; 0.002 wt.% Fe; 0.0003 wt.% Cu; 0.0003 wt.% Ni; 0.002 wt.% Pb; 0.002 wt.% Sn; 0.003 wt.% Zn;) used in this experiment was supplied by the Institute of Metal Research, Chinese Academy of Sciences. HP-Mg screws were machined by subtractive manufacturing method (Eontec, Dongguan, China) and used in rabbit experiments. The screws were 20 mm long, with a maximum outer diameter of 4 mm, a minimum diameter of 3 mm, a thread pitch of 1.5 mm and a thread width of 0.2 mm. The screw samples were ultrasonically cleaned with acetone, anhydrous ethanol and distilled water to remove residues and the screws were sterilized by 25kgy γ-rays. The raw material of PLA screws is poly L-lactic acid which was purchased from Chengdu Dikang Biomedical Company, China, and its size (Q-Φ4 × 20mm) is similar to that of Mg screws.

### Animals and experiments

A total of 14 skeletally matured male New Zealand White rabbits with mean weight 3.0 ± 0.5 kg were obtained from Songshan Lake Pearl Experimental Animal Technology Co., Ltd (Dongguan, Guangdong, China) and maintained with a 12-h light-dark cycle and constant temperature (25°C). All animal experiments were performed in accordance with ethical approvals from the Institutional Animal Care and Use Committee of Songshan Lake Pearl Animal Experiment Co., Ltd (SYSU-IACUC-2019-B091). All rabbits were randomly separated into three groups according to indicated time points: four rabbits for 4- and 8-week post-implantation, six rabbits for 16-week post-implantation. The rabbits were anaesthetized with 4% chloral hydrate (1.0 ml/100 g) and placed in the supine position. A 5-cm longitudinal lateral parapatellar incision was made across the knee. After the femoral condyle was exposed, an undersized drilling process (∼3.0 mm hole) was carried out perpendicular to the femoral shaft from the femoral lateral epicondyle. Each animal underwent the same surgical procedure on both knees, with a HP-Mg screw implanted in the right femoral condyle and a PLA screw implanted in the left femoral condyle, serving as the control. After implantation, the wound was sutured by layers after being washed and hemostasis. All rabbits were monitored carefully, and antibiotic (Amoxicillin 15 mg/kg) was administered subcutaneously once per day for three days post-operation to avoid post-operative infection. All the animals were allowed to move freely, no abnormal gait pattern was observed during the experiment. Upon 14 days and 7 days before harvesting the samples, 3 ml calcein green (10 mg/kg) and xylenol orange (90 mg/kg) were administered intramuscularly, respectively. Finally, femoral condyles with implanted screws were harvested at 4-, 8-, 16-week post-implantation for subsequent experiments. The peripheral soft tissues were removed carefully by surgical scissors. Specimens were fixed in 4% paraformaldehyde for 2 days and then preserved in 70% ethanol.

### Micro-CT analysis

Samples were fitted in the sample tube (Φ 37 mm) to process for micro-CT scanning (Viva CT 40, SCANCO MEDICAL, Brüttisellen, Switzerland). The parameters for X-ray settings were set as following: 70 kVp (Voltage), 114 μA (Current), 0.5 mm Al (Filter). For each specimen, the medial and lateral condyles were analyzed separately. Following the official user guidelines provided by SCANCO MEDICAL, the microstructural parameters were calculated by averaging the values obtained from five randomly selected regions of interest (ROIs) adjacent to the screw implant within epiphyseal trabecular region, each with a diameter of 3–4 mm. The parameters assessed included relative bone volume (BV/TV), bone mineral density (BMD), trabecular number (Tb. N), trabecular thickness (Tb. Th), trabecular separation (Tb. Sp) and connectivity density (Conn. D). To obtain the overall microstructural parameters for each specimen, the outcomes from both the medial and lateral condyles were averaged. A series of 2D tomographic images (2 5 0∼300 slices) along the direction parallel to the implant were segmented for 3D reconstruction of the peri-implant bone tissues. The parameters for CT-scan were set as following: high resolution (2048 × 2048 pixels) with 1000 projections, 18 μm (resolution), 2 5 0∼300 slices. Prior to 3D reconstruction, the resulting gray-images were segmented using a fixed threshold and a low-pass filter to minimize the noise (Sigma=1.2, support=2.0, threshold=184). The μCT system was pre-calibrated properly using phantom samples of known density to ensure accurate density readings.

### Gross observation and sample processing

Samples were transected along the central axis of screws into two parts by 300 CP Contact Point Sawing System (EXAKT Technologies, Inc., Oklahoma City, USA). Then macroscopic morphology was recorded. For each sample, one part was decalcified (9% formic acid solution) and used in histology and immunohistochemistry (IHC) staining. Prior to decalcification, the implant was removed carefully to better ensure the integrity of peri-implant bone tissues. Another part was undecalcified and used in hard tissue staining, histomorphometric and micromechanical analyses.

### Histology and immunohistochemistry staining

Decalcified samples were followed by dehydration and clearing and then were embedded in paraffin. Sections of 5 μm thick were cut and stained with hematoxylin and eosin (H&E). For staining, paraffin-embedded sections were first deparaffinized in xylene and rehydrated in diminishing concentrations of ethanol. Followed by incubation in 3% H_2_O_2_ for 10 min at room temperature to eliminate endogenous peroxidase activity. Antigen-retrieval was performed by heating the sections at 95°C for 6 min in sodium citrate/EDTA buffer (pH 6.0). Sections were cooled for 30 min at room temperature and washed with phosphate-buffered saline (PBS) for three times. Then sections were blocked with 5% BSA for 1 h at room temperature. RAM11 monoclonal antibody (Cat No. M0633, Dako, Glostrup, Denmark; 1:200 dilution) and transforming growth factor-β1 (TGF-β1) monoclonal antibody (Cat No. MA5-16949, Thermo Fisher Scientific, Waltham, USA; 1:200 dilution) were applied and incubated overnight at 4°C. After washing with PBST, sections were incubated with goat anti-rabbit IgG (HRP-labeled, Thermo Fisher Scientific; 1:1000 dilution) for 3 h at room temperature. Then the sections were re-washed with PBST and stained with DAB for 5 min. Finally, the sections were rinsed in distilled water, and counterstained with hematoxylin for 2 min. The expression intensity of the specific protein was evaluated by calculating the immunoreactive score (IRS) using the IHC Profiler plugin in ImageJ 1.53e (National Institutes of Health, USA) [54]. The ‘Cytoplasmic Stained Image’ mode and the ‘H DAB’ vector were selected for running the IHC Profiler. The IRS score was then determined by multiplying the staining intensity score (0–3) by the percentage of positively stained cells [55]. Data were presented as means of five serial slices. For each slice, 10 independent ROIs at 200× magnification were selected for quantitative analysis by three different observers. Images were captured using a Leica DM5500 fluorescent microscope at 50×, 100× and 200× magnifications.

### Hard tissue sectioning and staining

Undecalcified samples were embedded in methyl methacrylate and sectioned (about 2 0 0∼300 µm thick) by SP1600 Saw Microtome (Leica). The thick sections were further polished to 100 μm for Villanueva Bone staining (Sigma–Aldrich Co. LLC, USA) and measurement of bone formation and remodeling based on the staining of calcein green & xylenol orange. Under natural light and fluorescent light, Villanueva Osteochrome Bone Stain (Cat No. SHH0027, Sigma–Aldrich, USA) and fluorochromes stain were observed simultaneously by fluorescent microscope (DM5500, Leica).

### Micro-indentation test

Polished hard tissue sections were further used for micro-indentation test. Vickers–Hardness (VH) of peri-implant newly regenerated bones matrix (NRBM) and surrounding host bones (SHB) were detected by DUH-211S (Shimadzu, Kyoto, Japan), respectively [56]. Elastic Modulus and Bone Maturation Index (BMI) of peri-implant trabecular bones were further evaluated [57]. BMI (%) was calculated the ratio between VH of NRBM and SHB [57].

### Scanning electron microscopy imaging

Polished hard tissue sections were acid etched (37% Phosphoric acid) for 20 s and followed by bleaching (4.00–4.99% chlorine basis sodium hypochlorite solution, Sigma–Aldrich, USA) for 20 min at room temperature. Then the sections were rinsed in distilled water and sequentially dehydrated (70%, 80%, 90% ethanol for 20 min, and absolute ethanol for 1 h) [58]. Prior to scanning, sections were firstly coated with a thin layer of gold by sputter coater (SPI Supplies, USA). Quanta 200 SEM (FEI Co., USA) was used to observe peri-implant trabecular bones and osteocytes. In addition, both secondary electron (SE) imaging and back-scattered electron (BSE) imaging were conducted.

### Calculation of peri-implant bone area ratio (%) and bone-to-implant contact ratio (%)

ROI defined for calculating peri-implant bone area was composed of area enclosed by adjacent threads (as blue line indicated in Figure 5B). Specifically, the peri-implant bone area ratio was calculated for each pitch individually, and then the average was calculated to represent the overall peri-implant bone area ratio for each specimen. The length of the ROI for each pitch equaled to 1 mm (approximately twice the pitch depth) as previously reported [59, 60]. Trabecular bone area within ROI and total area of ROI were determined by using Image J 1.53e (National Institutes of Health, USA). The peri-implant bone area (%) was calculated using the following equation: Peri-implant Bone Area Ratio=Trabecular Bone Area within ROI/Total Area of ROI × 100%. In addition, the bone-to-implant contact ratio (%) was calculated using the following equation: Bone-to-implant Contact Ratio=the length of bone contact with implant surface/the length of the implant surface × 100%.

### Finite element model used for calculation of stress distribution around the bone tunnel

The 3D models of our tested screws were created using SolidWorks (version 2016, SolidWorks Corp., Waltham, MA, USA). The 3D model of the Mg screw fixed into the trabecular bone was imported into ABAQUS workbench (Hibbit Inc., Rhode Island, USA) to calculate the stress distributions [10]. The finite element model covered two parts including the Mg screw and the simplified trabecular bone. A finite element model with 10-node quadratic tetrahedral elements (C3D10) was used. The Mg screw generated a total of 14 168 elements, while the trabecular bone created 28 530 elements (Supplementary Figure S2). In this model, Young’s modulus, E = 17 GPa and Poisson ratio, ν = 0.26, were used for the trabecular bone, while E = 45 GPa and ν = 0.35 for the Mg screw and E = 2.7 GPa and ν = 0.4 for PLA screw were used according to our published data [61]. Afterwards, a reference point and a force were set to conduct finite element simulation for calculation of the stress distribution around the bone tunnel.

### Statistical analysis

All statistical data were expressed as mean±SD. Statistical significance comparing two groups with non-parametric data was assessed by nonparametric test (Mann–Whitney *U*-test) with GraphPad Prism 10 software (GraphPad Software Inc., La Jolla, CA, USA). For all comparisons, *P*-values <0.05 were considered statistically significant.

## Results and discussion

### High-purity Mg screws improved the microstructural characteristics of peri-implant bones

**Figure 1.**
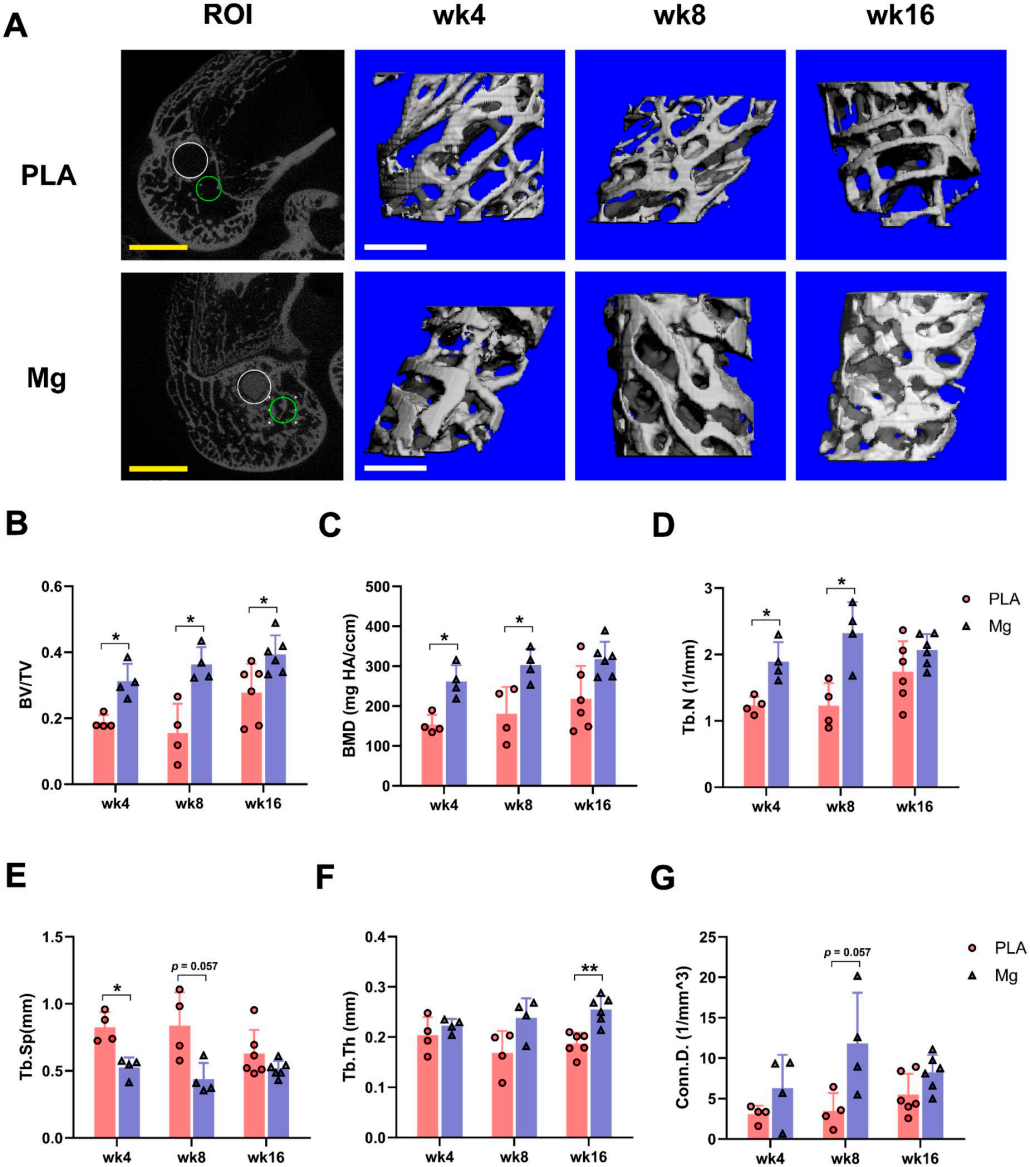
High-purity Mg screw improved peri-implant bone microstructural characteristics. (**A**) Representative ROI of peri-implant trabecular bone tissues at the sagittal plane (left panel) and corresponding 3D reconstruction (right three panels). The large circle indicated the region where the implant was located. The small circle represented the individual ROI for following morphometric analyses. Scale bar in the 2D images = 5 mm; Scale bar in the 3D images = 1 mm. (**B–G**) Microstructural properties of peri-implant bone tissues (*n* = 4 for weeks 4 and 8; *n* = 6 for week 16). BV/TV, relative bone volume (%); BMD, bone mineral density (mg HA/ccm); Tb. N, trabecular number (1/mm); Tb. Sp, trabecular separation (mm); Tb. Th, trabecular thickness (mm); conn. D, connectivity density (1/mm^3^). *P <* *0.05, **P <* *0.01.

The gross morphology of PLA screw and HP-Mg screw for following implantation was shown in Supplementary Figure S2A and B. The basic structure parameters of the screws were quantified as a length of 20 mm, a major diameter of 4 mm, a minor diameter of 3 mm, a thread pitch of 1.5 mm, a thread width (crest) of 0.2 mm and a buttress thread shape. After harvesting the specimens at indicated time points, μCT analysis was performed first. The representative cross-sections of rabbit distal femur in different groups were shown in Figure 1A (left panel). The ROI for following peri-implant bone morphometric analyses was outlined by the green circle located in region at a comparable distance and direction from the implant (Figure 1A). 3D reconstructed images of peri-implant trabecular bones at different time points were presented in Figure 1A (Right three panels). Significant increase of peri-implant bone mass was observed in Mg group as compared to PLA group. The quantitative outcomes of peri-implant bone microstructural properties were summarized and presented in Figure 1B–G. The microstructural parameters of peri-implant trabecular bone tissues supported our hypothesis that HP-Mg screw improved quality of peri-implant bone tissues compared to PLA screw with significantly higher level of BV/TV, BMD, Tb.N and Conn.D and lower level of Tb.Sp at 4- and 8-week post-implantation. By 16-week post-implantation, the differences in the microstructural parameters between the Mg and PLA groups had narrowed, reaching comparable levels. Longitudinal comparison indicated that the microstructural parameters of the two groups both maintained an increasing trend over time.

Compared to the bioinert PLA screw [62], the beneficial effects of Mg screws on the microstructural characteristics of peri-implant bone tissues can be primarily attributed to the osteo-promotive properties of implant-derived Mg ions [7]. These ions exert their influence through both direct and indirect mechanisms. Firstly, Mg ions directly stimulate the osteogenic differentiation of mesenchymal stromal cells [63]. Secondly, Mg ions indirectly facilitate osteogenesis by modulating the neurological, vascular and immune microenvironment around the implant site. For example, implant-derived Mg ions were demonstrated to induce local neuronal production of calcitonin gene-related peptide (CGRP) to improve not only the osteogenic differentiation of periosteum-derived stem cells but also the angiogenesis during fracture healing [12, 64, 65]. Furthermore, recent study has explored the central role of immunomodulatory macrophages in the formation of a pro-osteogenic immune microenvironment in response to Mg ions [66].

### Gross-view and microscopic morphology of peri-implant bone tissues

**Figure 2.**
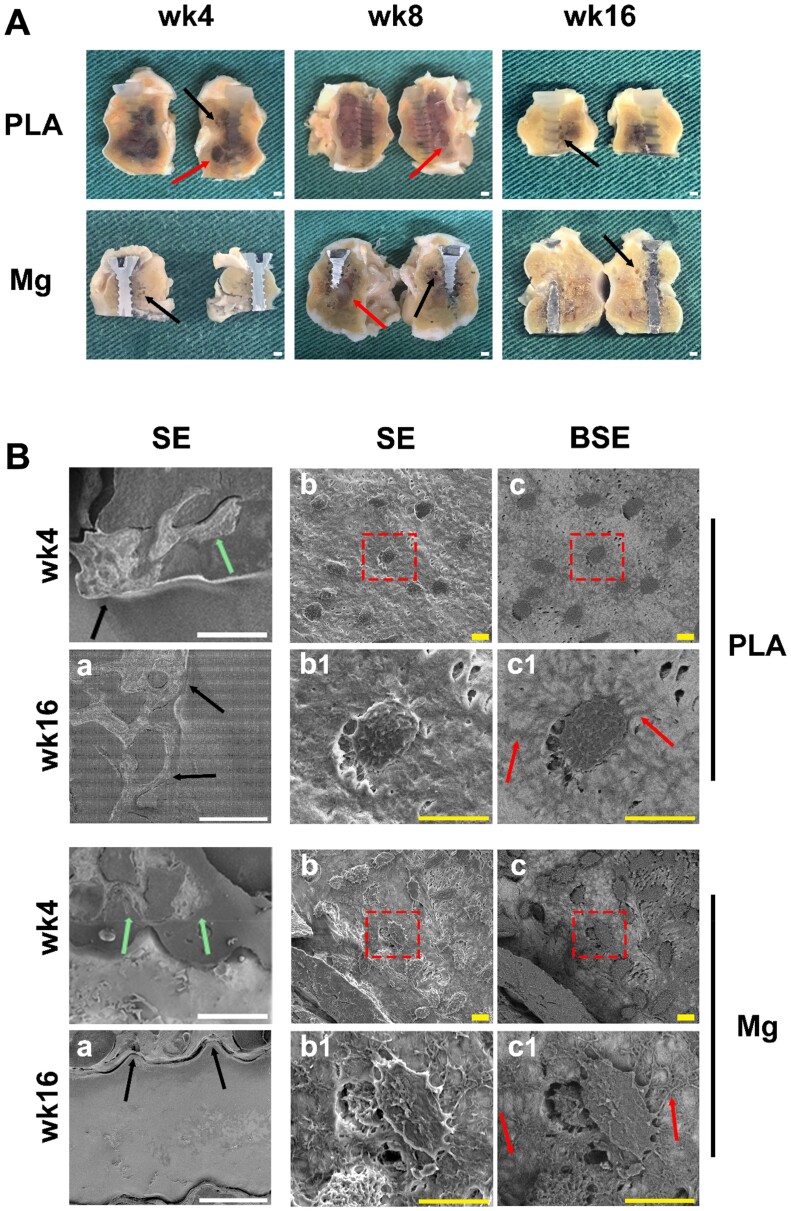
Gross and microscopic morphology of peri-implant bone tissues. (**A**) Gross observation of peri-implant bone tissues. Arrows indicated cavities; scale bar = 1 mm. (**B**) Microscopic observation of peri-implant bone tissues by SEM. Peri-implant bone tissues at 4- and 16-week post-implantation were shown in the leftmost panel. Peri-implant bone tissues in panel *a* were magnified into panels *b* and *c* by SE and BE, respectively. Osteocytes within the dotted box in panels b and c were further magnified into panels b1 and c1, where the arrows indicated lacuno-canalicular network architecture formed by osteocytes located at peri-implant bone tissues; scale bar in panel A = 1 mm; scale bar in panel B = 10 μm. SE, secondary electrons imaging; BSE, back-scattered electrons imaging.

After sagittal dissection of femur condyles, the matrix-screw interface in both the Mg and PLA groups was comparable. Of note, the neighboring porosity was both observed in the Mg and PLA group, which manifested as the occurrence of neighboring smaller (as black arrows indicated) or even larger cavities (as red arrows indicated) (Figure 2A). In Mg group, the peri-implant cavities may be attributed to the accumulation of hydrogen gas during degradation [35]. Nevertheless, the peri-implant cavities in Mg group did not further develop as the degradation progressed. Notably, the peri-implant cavities in PLA group were comparable to Mg group (Figure 2A). Based on literature review, there are several potential triggers for the impairments on peri-implant bone tissues in PLA group, including incomplete ossification, inflammatory osteolysis and FBRs [43, 49, 67, 68]. The degradation of Mg screw characterized with reduced screw volume and ingrowth of peri-implant bone tissues was observed since 4-week post-implantation, while no obvious changes were observed in PLA group even at 16-week post-implantation, suggesting the faster *in vivo* degradation rate of HP-Mg screw compared to PLA screw, well in line with previous studies [35, 52]. Since the degradation of the implants may impair their mechanical integrity, the degradation behavior of the implants should be optimal when it matches the healing process of the bone defect or fracture. Compared to PLA screw, HP-Mg screw possesses comparable mechanical strength to cortical bone tissue, which not only alleviate the stress-shielding effect but also provide stronger mechanical support [7]. When compared the degradation behavior of Mg-based materials and polymer-based materials *in vivo* according to literature review (Supplementary Figure S1) [7, 36], we can notice that, although the *in vivo* degradation rate (the slope of the curve) of HP-Mg screw is much higher than PLA screw, the duration of functional phase is much longer for HP-Mg screw than that for PLA screw, which contribute to the much stronger mechanical support during early-stage healing process.

Microscopically, peri-implant bone tissues in close contact with screw interface were both observed in PLA group and Mg group at 4- and 16-week post-implantation by SEM scanning (Figure 2B). At 4-week post-implantation, bone-to-implant contact was less frequently observed in the Mg group (as black and green arrows indicated; Figure 2B), possibly due to the faster degradation of Mg screws and the consequent release of hydrogen gas. However, bone-to-implant contact was commonly observed in both Mg and PLA groups at 16-week post-implantation. Besides, the density of osteocytes locating at peri-implant trabecular bones looked similar in different groups (Figure 2B, b and c). The osteocyte LCN architecture was further visualized by BSE imaging as previously described [69]. As compared to PLA screw, osteocytes locating at peri-implant regions held significantly higher number of dendrites and more intact LCN architecture in Mg group, which was manifested as denser dendrites and intercellular crosslinks (as red arrows indicated; Figure 2B, b1 and c1) [46]. LCN architecture is formed through dendritic processes and contributes to forming strong integrin attachments which plays important roles in bone remodeling as mechanotransducers which transmit mechanical signals from dendrites to the cell body [70–72]. In general, a well-preserved and interconnected LCN architecture is associated with regenerated normal bone matrix and functionality [73]. Thus, more intact osteocyte LCN architecture in Mg group implied enhanced new bone formation and its functionality, such as cell–cell communication, nutrients exchange and mechanotransduction.

### High-purity Mg screws reinforced peri-implant bone remodeling

**Figure 3.**
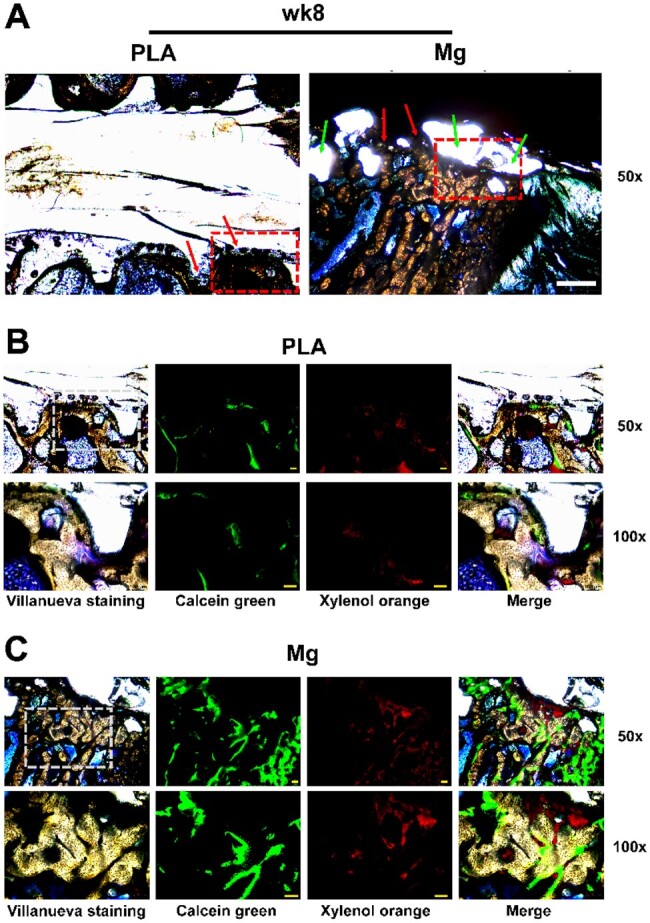
High-purity Mg screw reinforced peri-implant bone remodeling at week 8 post-implantation. (**A**) Representative images of peri-implant tissues stained with villanueva bone stain at week 8 post-implantation. ROI was delineated by a dotted rectangle; scale bar in panel A = 1 mm. (**B** and **C**) Representative images of ROI stained with villanueva osteochrome bone stain and calcein green/xylenol orange fluorochromes stain at week 8 post-implantation. Regions within the dotted box were further magnified; scale bar = 100 μm.

To monitor peri-implant bone remodeling dynamics, villanueva osteochrome bone staining and calcein green/xylenol orange fluorochromes staining were conducted [74]. Under natural light, mineralized bone and osteoid can be observed in orange and jade green/dark green, respectively. Under the fluorescent light, mineralizing surfaces at different time points that stained with calcein green and xylenol orange can be observed. Obvious bone-to-implant contact was observed in both Mg and PLA group at 8- and 16-week post-implantation (as red arrows indicated; Figure 3A; Supplementary Figures S3 and S4A). However, cavities/bubbles without bone ingrowth were also observed in Mg group at all the three time points (as green arrows indicated; Figure 3A; Supplementary Figures S3 and S4A), which might be originated from the release of hydrogen gas during Mg degradation. Qualitatively, representative images suggested that the amount and size of peri-implant cavities appeared to decrease at 8- and 16-week post-implantation compared to 4-week post-implantation in the Mg group (as indicated by green arrows; Figure 3A; Supplementary Figures S3 and S4A). Furthermore, acellular calcified matrix layer which was labeled by fluorescence was also observed accompanied by Mg degradation (as indicated by red-dashed circle; Supplementary Figure S4C), echoing the previous study about the degradation of Mg alloy implant [74]. In addition, fluorochromes staining showed that there were apparently more mineralizing surfaces in Mg group at all time points, suggesting that the degradation of Mg screw showed bioactive impact on peri-implant bone remodeling (Figure 3B and C; Supplementary Figures S3 and S4B and C). Thus, as compared to PLA screws, HP-Mg screws appeared to enhance peri-implant bone remodeling, accompanied by the formation of limited and localized peri-implant cavities. Especially, the peri-implant cavities in the Mg group did not progressively enlarge over time.

### High-purity Mg screws improved biomechanical properties of peri-implant trabecular bones at matrix level

**Figure 4.**
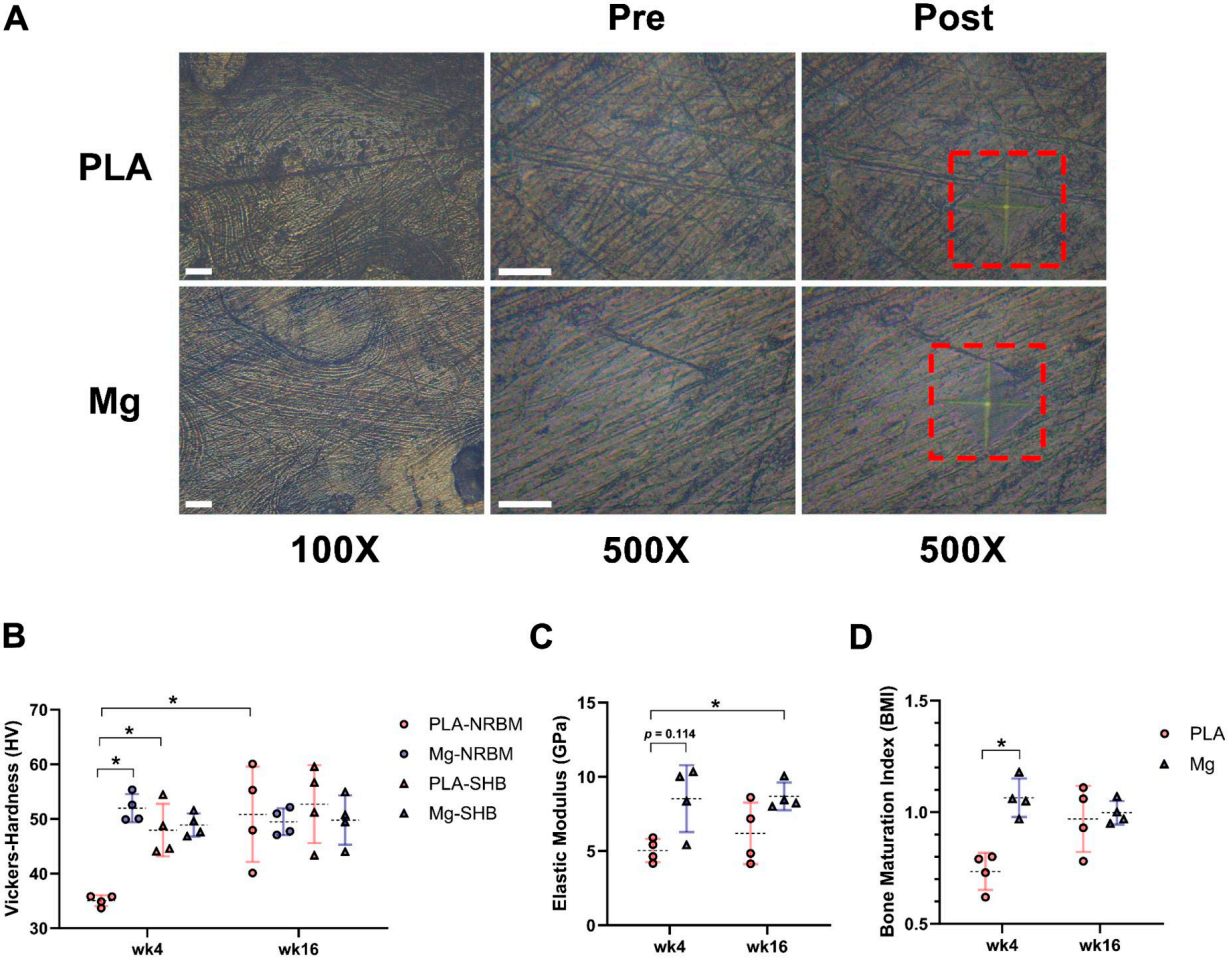
High-purity Mg screw improved biomechanical properties of peri-implant trabecular bones at matrix level. (**A**) Representative images of peri-implant trabecular bone tissues under the microscope of microhardness tester pre- and post-indentation. Dashed boxes showed the indentation area with diagonal lines; scale bar = 100 μm. (**B**) Vickers–Hardness of peri-implant newly regenerated bone matrix (NRBM) and surrounding host bones (SHB) at week 4 and 16 post-implantation (*n* = 4). (**C**) Elastic modulus of peri-implant trabecular bones at week 4 and 16 post-implantation (*n* = 4). (**D**) Bone maturation index (BMI) of peri-implant trabecular bones at week 4 and 16 post-implantation (*n* = 4). *P <* *0.05.

In addition to peri-implant bone histomorphometric properties, we further evaluated the biomechanical properties of peri-implant tissues at matrix level by measuring the VH and Young’s modulus of peri-implant NRBM and SHB, respectively (Figure 4A). The BMI was compared by calculating the ratio between VH of NRBM and SHB. Consequently, Mg screws improved the microhardness, elastic modulus and BMI of peri-implant NRBM at 4-week post-implantation to the level of SHB (Figure 4B–D). At week 16 post-implantation, the microhardness, elastic modulus and BMI of peri-implant newly restored bones in Mg group and PLA group all remained at the comparable level (Figure 4B and C). Thus, HP-Mg screws significantly elevated the biomechanical properties of peri-implant trabecular bones at an early-stage post-implantation.

Bone hardness, a critical property strongly correlated with the degree of bone matrix mineralization, determines bone strength at the tissue level [75]. Previous studies have shown that newly regenerated osteons have lower hardness than older mineralized osteons [76]. Although Mg^2+^ is widely used to promote bone growth and regeneration, it can also chemically inhibit the mineralization process [77]. Recent research has explored the crucial role of Mg ions in bone biomineralization during embryonic development, demonstrating that appropriate regulation of Mg^2+^ concentration is vital for bone biomineralization [78]. Interestingly, our study revealed that HP-Mg screws significantly accelerated peri-implant bone maturation compared to PLA screws. Specifically, the maturation index of newly formed bone tissues in the Mg group reached the level of mature osteons at 4-week post-implantation, while the maturation index in the PLA group was considerably lower than that of mature osteons at the same time point (Figure 4D). Collectively, HP-Mg screws not only facilitate bone formation but also accelerate bone maturation compared to PLA screws.

### High-purity Mg screws improved the ingrowth of peri-implant trabecular bones into implant pitches

**Figure 5.**
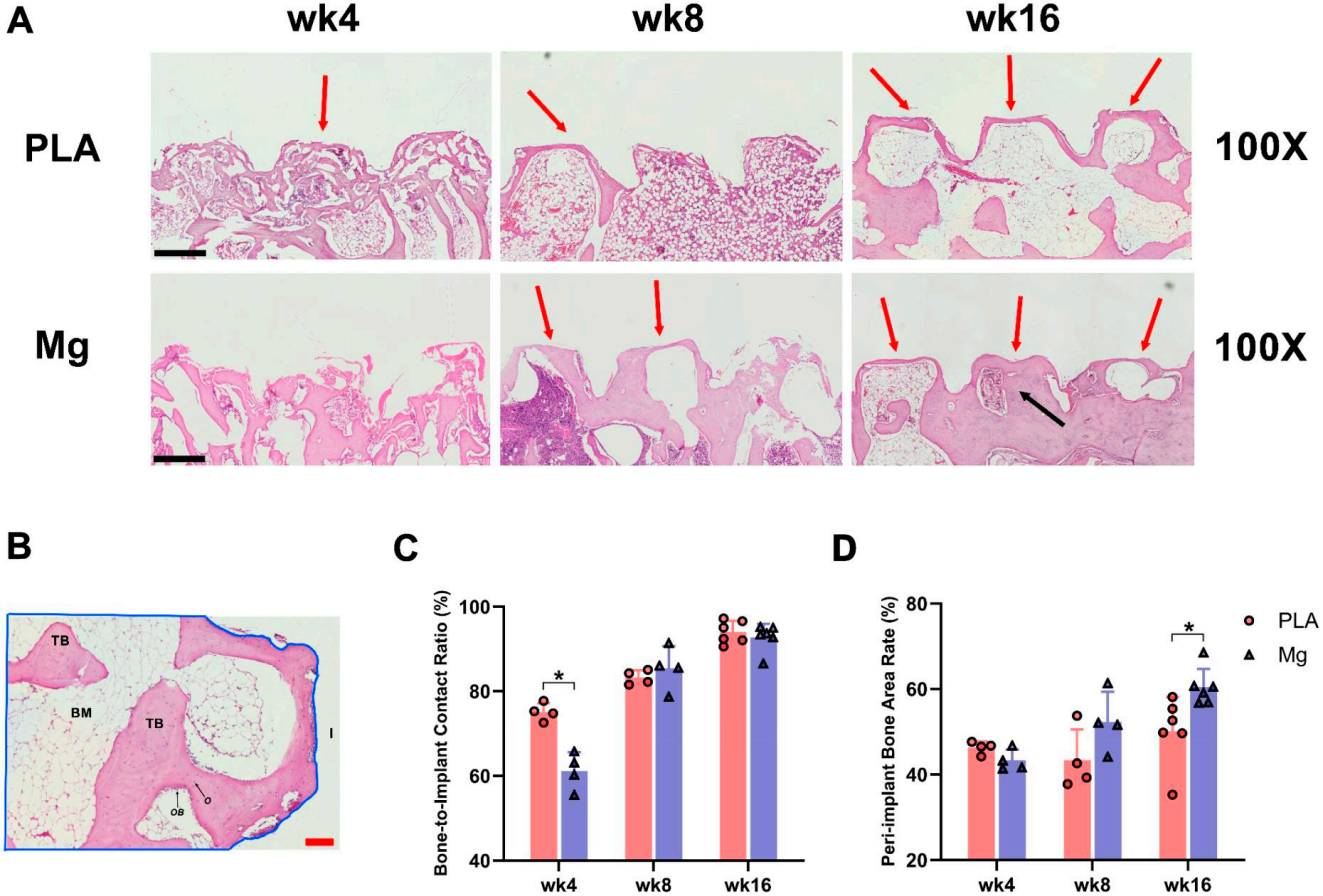
High-purity Mg screw improved the ingrowth of peri-implant trabecular bones into implant pitches. (**A**) H&E stain of peri-implant trabecular bone tissues. The arrows indicated bone-implant contact; Mg screw pitch that was filled with trabecular bones; scale bar in panel A = 500 μm. (**B**) ROI for calculating peri-implant bone area was enclosed by lines. TB, trabecular bone; BM, bone marrow; O, osteocyte; OB, osteoblast; I, implant; scale bar in panel B = 100 μm. (**C**) Comparisons of bone-to-implant contact ratio (%) at week 4, 8 and 16 post-implantation (*n* = 4 for weeks 4 and 8; *n* = 6 for week 16). (**D**) Comparisons of peri-implant bone area (%) at week 4, 8 and 16 post-implantation (*n* = 4 for weeks 4 and 8; *n* = 6 for week 16). *P <* *0.05.

The histological morphology of peri-implant bone tissues was observed by H&E staining (Figure 5A) and the bone-to-implant interface was further evaluated as previously reported [79–81]. We found that the bone-to-implant contact rate (%) was significantly reduced in Mg group at 4-week post-implantation as compared to that in PLA group, suggesting the suppressive effects of Mg degradation on peri-implant bone ingrowth at an early-stage post-implantation, which might be caused by longer matrix-screw distance and accumulation of hydrogen gas accompanied by degradation (Figure 5C). Nonetheless, the bone-to-implant contact rate in the Mg group recovered to a level comparable to that of the PLA group at both 8- and 16-week post-implantation (Figure 5C). Such results were also consistent to the above observations obtained by SEM (Figure 2B). Of note, the screw pitch in Mg group at 16-week post-implantation was observed to be filled with *de novo* formed spongy bones with fewer deposition of adipose tissues (as black arrow indicated; Figure 5A). We further measured the peri-implant bone area ratio (%) to comprehensively evaluate the degree of trabecular bone ingrowth (Figure 5B and D). Interestingly, there was no significant difference in the peri-implant bone area ratio, which primarily reflects the bone mass within the peri-implant region, at 4- and 8-week post-implantation between the Mg and PLA group. Such discrepancy between the bone-to-implant contact rate and the peri-implant bone area ratio suggests that, although the faster degradation of Mg screw and the subsequent release of hydrogen gas may result in a lower bone-to-implant contact rate at 4-week post-implantation by inhibiting the NRBM from contacting the implant surface, they do not adversely affect bone regeneration process. Mg screws significantly improved the peri-implant bone area at 16-week post-implantation in contrast to PLA screws (Figure 5D). Such results were consistent with the activated peri-implant bone regeneration observed in Mg group (Supplementary Figure S4). After 8-week post-implantation, apparently denser peri-implant trabecular bones were formed in Mg group, while adipocyte-like structures were primarily deposited in PLA group (Figure 5A). The inhibitory effect of Mg screws on marrow adipose deposition was noticed, whereas further studies are required to explore its underlying mechanisms. Disturbed bone-adipose balance in marrow contributed to reduced bone mass and increased marrow adipose tissue (MAT) accompanied by aging and obesity [82–84]. Notably, Mg^2+^ deficiency could affect the bone-adipose balance not only by increasing adipogenesis due to alterations in bone homeostasis, but also by raising oxidative stress, elevating inducible nitric oxide synthase and H_2_O_2_ production [83, 85–89]. Besides, synergistic effects of Mg ions and simvastatin were also reported to compromise cholesterol synthesis under high-fat diet in mice, which in turn suppressed marrow fat deposition and bone resorption [90]. CGRP, which was secreted excessively by dorsal root ganglia under Mg stimulation and promoted osteogenic differentiation of PDSCs in rats, inhibited adipogenic differentiation of BMSCs as well [12, 91].

### High-purity Mg screws alleviated macrophage infiltration at an early-stage post-implantation

**Figure 6.**
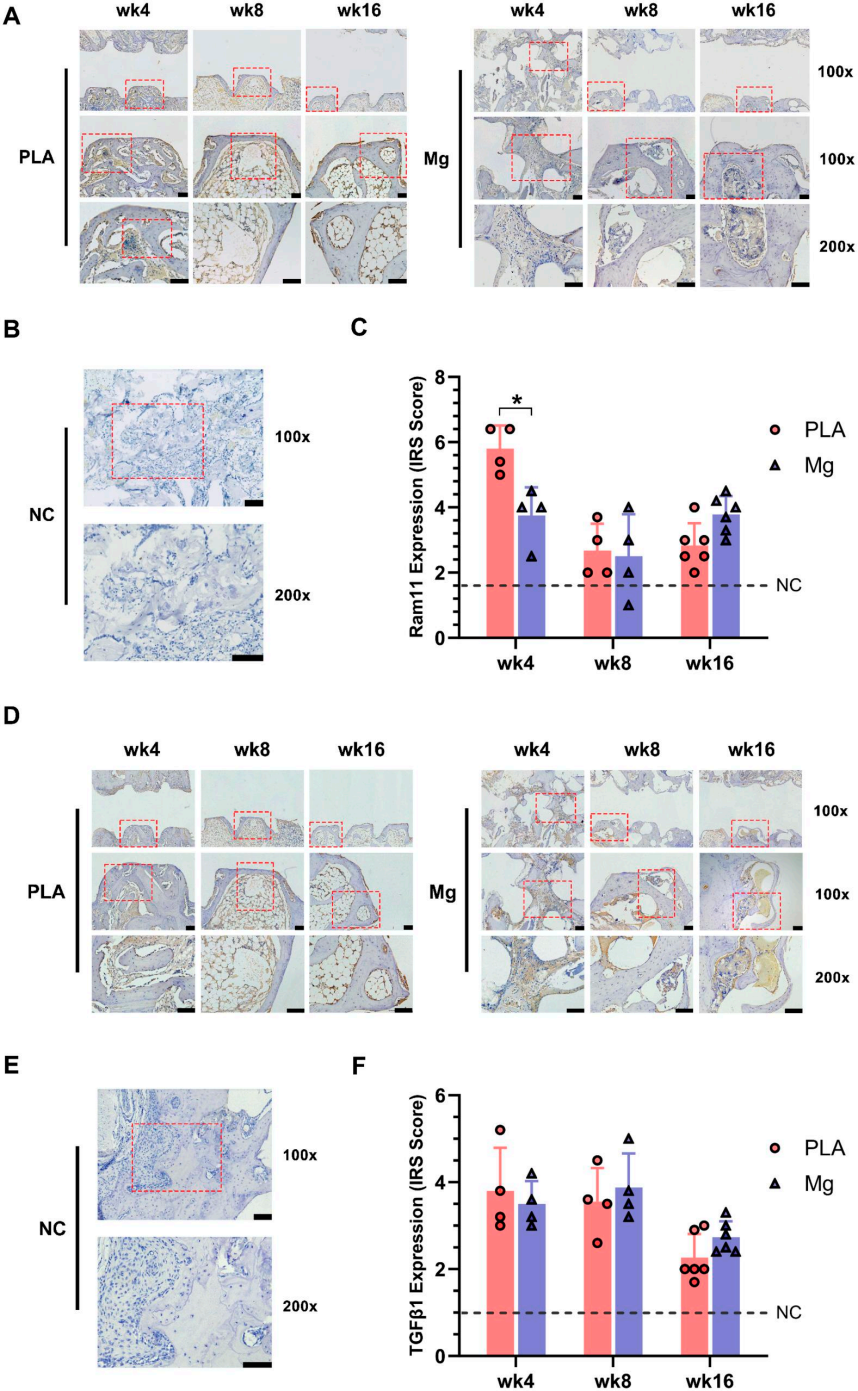
High-purity Mg screw alleviated macrophage infiltration at early stages post-implantation. (**A**) Representative images of peri-implant trabecular bones by IHC staining targeting at macrophage-specific RAM11; regions within the dashed boxes were recognized as ROI and further magnified. (**B**) Representative images of IHC negative control. NC, negative control. (**C**) The IRS-based expression intensity of RAM11 at peri-implant bone regions (*n* = 4 for weeks 4 and 8; *n* = 6 for week 16). (**D**) Representative images of peri-implant trabecular bones by IHC staining targeting at TGF-β1; regions within red-dashed boxes were recognized as ROI and further magnified. (**E**) Representative images of IHC negative control. NC, negative control. (**F**) The IRS-based expression intensity of TGF-β1 at peri-implant bone regions (*n* = 4 for weeks 4 and 8; *n* = 6 for week 16). IRS, immunoreactive score. Scale bar = 100 μm. *P <* *0.05.

Since both HP-Mg and PLA screw were reported to cause FBR, which is associated with peri-implant fibrosis [53, 92], the infiltration of macrophages was investigated by using macrophage specific RAM11 antibody to reflect the degree of inflammatory responses after implantation. Higher expression of RAM11 was observed in PLA group at 4-week post-implantation (Figure 6A–C), suggesting the severer FBR at an early stages post-implantation in PLA group compared to Mg group. For later stages post-implantation, there was no difference between Mg and PLA group (Figure 6A–C). So far, the crucial role of Mg in immunomodulation has been widely explored. For example, Mg deficiency was demonstrated to be implicated in chronic inflammation [93], while Mg ions can induce the formation of a pro-osteogenic immune microenvironment via stimulating the production of inflammatory cytokines in macrophages [66]. Furthermore, Mg-based implant materials have been shown to induce faster inflammation resolution and improve tissue repair by promoting the production of M2 profiles in macrophages [94]. Thus, our results further supported that Mg screws accelerate inflammation resolution compared to PLA screws at the early-stage post-implantation.

Since excessive infiltration of macrophages has been demonstrated to impair bone regeneration and contribute to fibrous nonunion in fracture healing [95], we further assessed the presence of fibrous tissues within peri-implant regions, particularly in the PLA group. As H&E staining showed, there was no peri-implant fibrosis in both PLA and Mg group at all time points (Figure 5A). To further reinforced above observation, we evaluated the expression of TGF-β1, which was a master regulator of tissue fibrosis produced predominantly by macrophage [96]. It turned out that the expression of TGF-β1 in peri-implant bone tissues showed no difference between Mg and PLA groups at all time points (Figure 6D–F). Additionally, we observed that the expression of TGF-β1 was higher in both the Mg and PLA groups at weeks 4 and 8 post-implantation compared to that at week 16. Our observations were consistent to existing research conclusion that higher level of TGF-β1 expressed at sites with active osteogenesis and angiogenesis, such as fracture callus [97–100]. Meanwhile, decreased TGF-β1 at 16-week post-implantation reduced the risk of fibrosis or heterotopic ossification as well [101–103]. Collectively, above results suggested that Mg screw was associated with alleviated FBRs without inducing peri-implant fibrosis [97, 104].

### High-purity Mg screws can effectively reduce stress concentration around the bone tunnel surface

**Figure 7.**
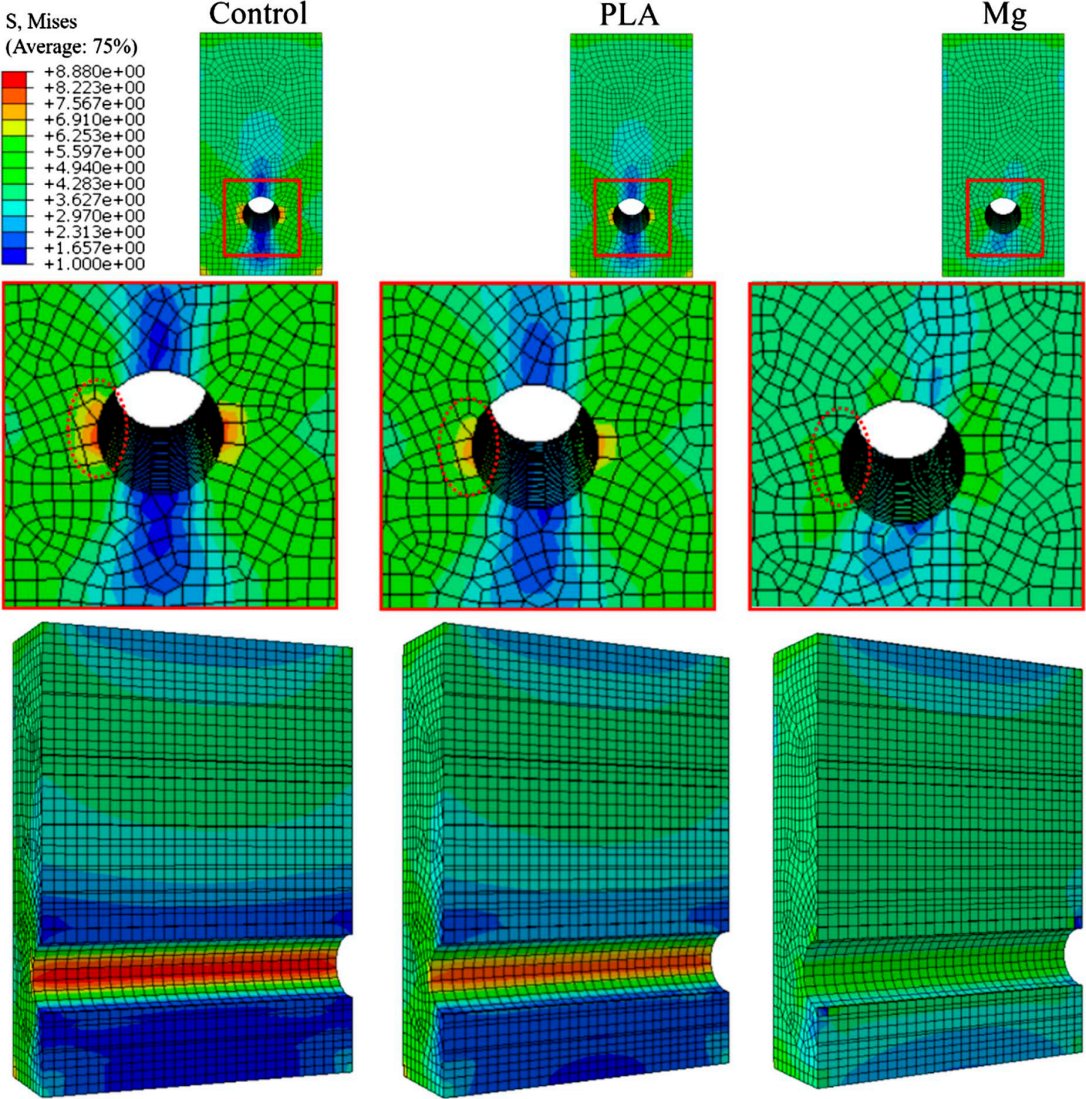
Three numerical simulation models with or without the insertion of the PLA or Mg screw exhibiting the stress distribution around the peri-implant bone tunnels.

We then performed FEA to compare the stress distribution around the peri-implant bone tunnel between Mg and PLA screws. We found that the creation of bone tunnel may alter the stress distribution once a force is applied. The insertion of implants with elastic modulus close to bone tissue may reduce the abnormal deformation in the non-homogeneous model in a load-bearing condition. As depicted in Figure 7, the empty peri-implant bone tunnel caused higher stress located at both medial and lateral sides around the tunnel surface. The implantation of PLA screws effectively reduced the stress concentration, showing aa lower maximum von Mises stress from 8.87 to 8.20 MPa. Encouragingly, compared to the PLA screws, the Mg screws further homogenized the spatial stress distribution, exhibiting a maximum von Mises stress with only 6.30 MPa, suggesting gradual adaptation of homogeneous stress distribution around the peri-implant bone tunnel surfaces (Figure 7). From the FEA analysis, the Mg metals exhibited an elastic modulus close to the natural bone, indicating less concerns on non-homogeneous stress distribution around the bone fractures or defects after the use of the Mg-based implants. For those patients especially suffering from severe osteoporosis, the homogeneous stress distribution may reduce the risks of trabecular microfractures, suggesting the better biomechanical compatibility of Mg screw over PLA screw.

## Conclusions

The present study evaluated the local effects of HP-Mg screw on the peri-implant trabecular bone as compared to commercial biodegradable PLA screw in rabbit distal femur. As a result, significantly enhanced peri-implant bone quality and alleviated inflammatory response were observed in Mg group at an early-stage post-implantation. As compared to PLA screw, HP-Mg screw promoted peri-implant bone ingrowth with higher biomechanical properties, more mature osteocyte LCN architecture and accelerated bone remodeling process. Post-implantation complications such as FBRs were relieved in Mg group as well. Besides, neither exacerbated gas cavities nor fibrosis was observed after 16-week post-implantation. In addition, FEA indicated that HP-Mg screw presented more homogeneous stress distribution around implant that reduced the risks of trabecular microfractures and implant loosening. Our findings laid the foundation for the potential use of HP-Mg screws as fixators of bone defects or fractures at the non- or partially load-bearing sites, such as hallux valgus, patellar fracture, ligament reconstruction and distal radius fracture. Collectively, HP-Mg screw has shown promising potential as an alternative to polymer screw in various clinical applications.

## Supplementary Material

rbae095_Supplementary_Data

## Data Availability

The full data of the research are contained in this article and the supplementary material.
