# Supplementary material for: A comparative study on the effects of biodegradable high-purity magnesium screw and polymer screw for fixation in epiphyseal trabecular bone
Source: Regen Biomater. 2024 Sep 3;11:rbae095. doi: 10.1093/rb/rbae095 (PMC11427752; doi:10.1093/rb/rbae095)
Supplement: rbae095_Supplementary_Data [file rbae095_supplementary_data.zip › Supporting Information_final.docx]

**Supporting Information**


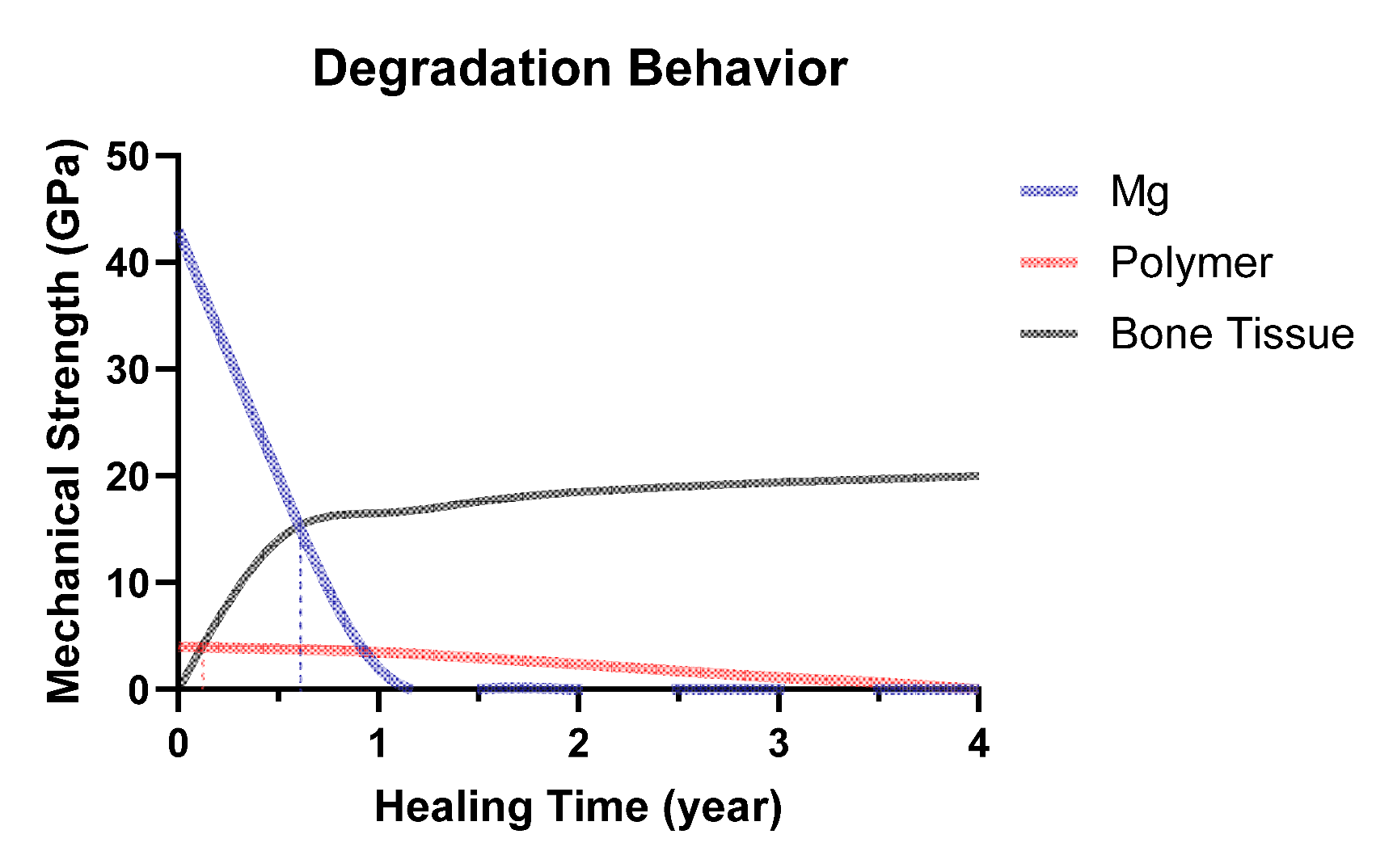


**Supplementary Figure 1.** Schematic diagram illustrating the *in vivo* degradation behavior of magnesium-based and polymer-based materials according to literature review. The blue dashed line indicates the time point at which magnesium-based implants transition from the functional phase to the absorption phase. Similarly, the red dashed line marks the time point at which polymer-based implants shift from the functional phase to the absorption phase.


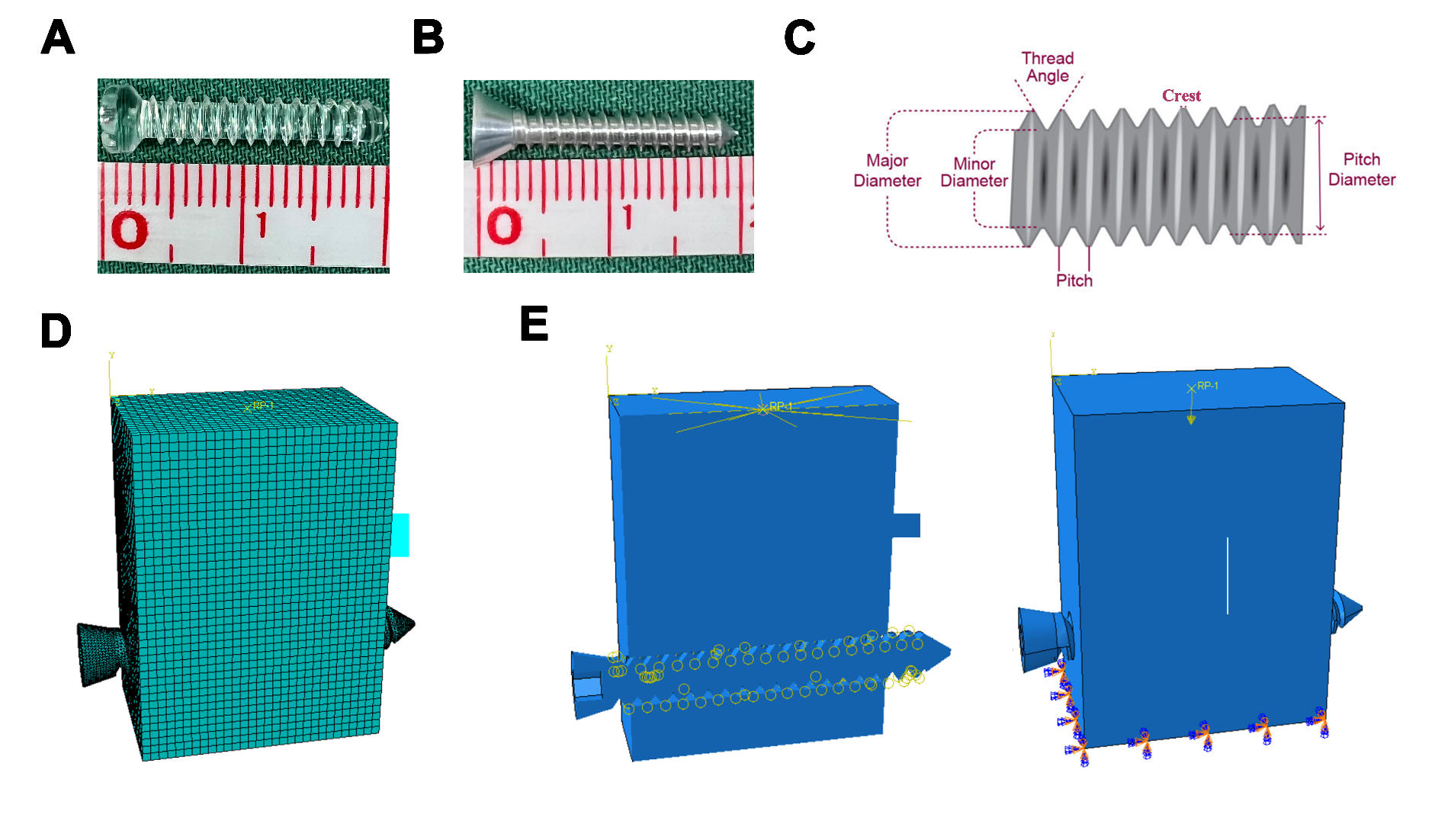


**Supplementary Figure 2.** Gross morphology of screws and a simplified trabecular bone model fixed by the screw for finite element simulation. (A and B) Gross morphology of PLA screw (A) and high-purity Mg screw (B). (C) The key parameters applied in the screw for the finite element model. (D) Images of finite element mesh for the trabecular bone fixed by the screw. (E) The applied boundary conditions and defined interactions for simulation.


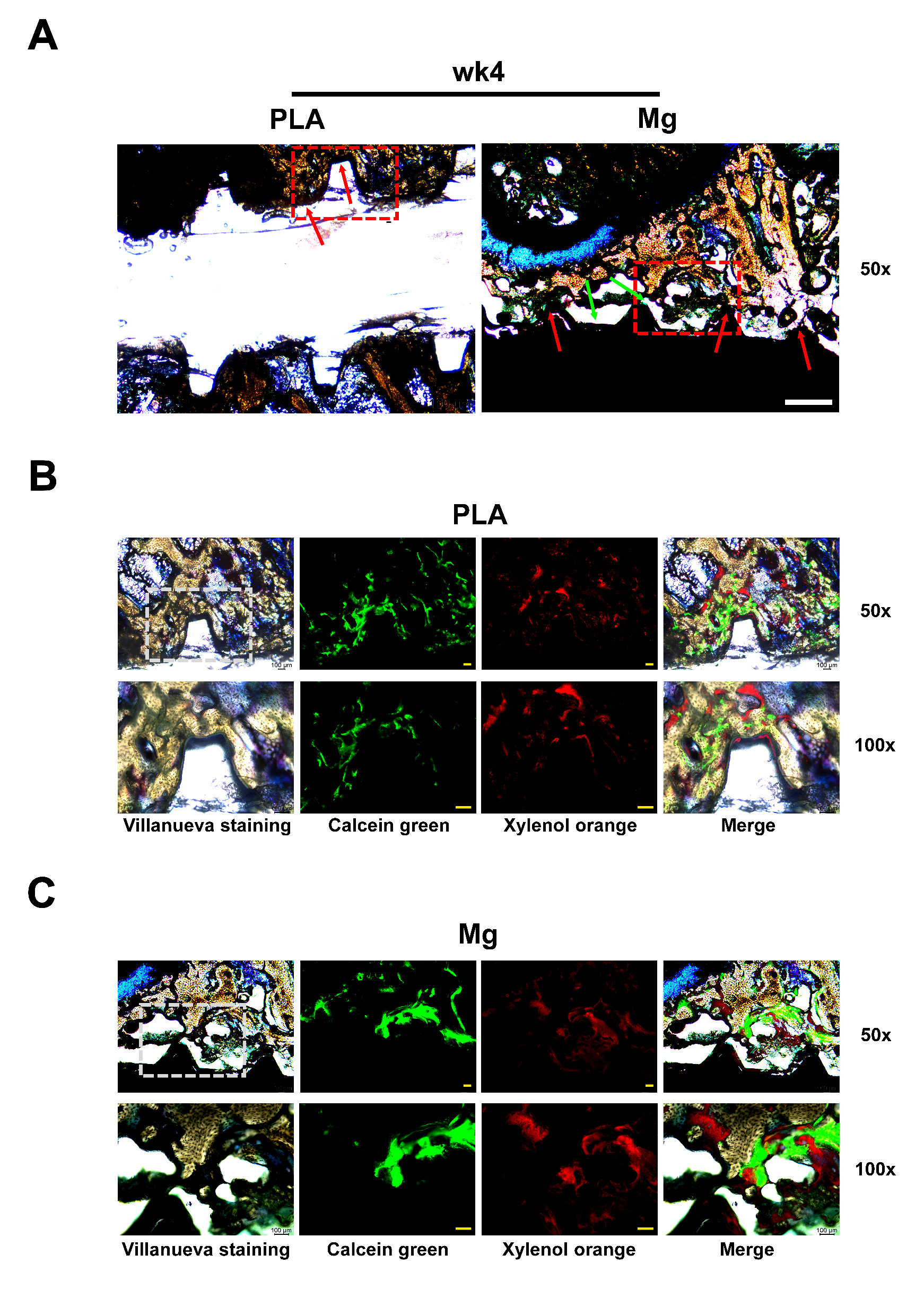


**Supplementary Figure 3.** High-purity Mg screw improved peri-implant bone remodeling at week 4 post-implantation. (A) Representative images of peri-implant tissues stained with Villanueva bone stain at week 4 post-implantation. Red arrows indicated bone-implant contact; green arrows indicated gaps between bone and implant; ROI was marked by a red dashed rectangle; white scale bar = 1 mm. (B and C) Representative images of ROI stained with Villanueva osteochrome bone stain and calcein green/xylenol orange fluorochromes stain at week 4 post-implantation. Regions within the white dashed box were further magnified; scale bar = 100 μm.


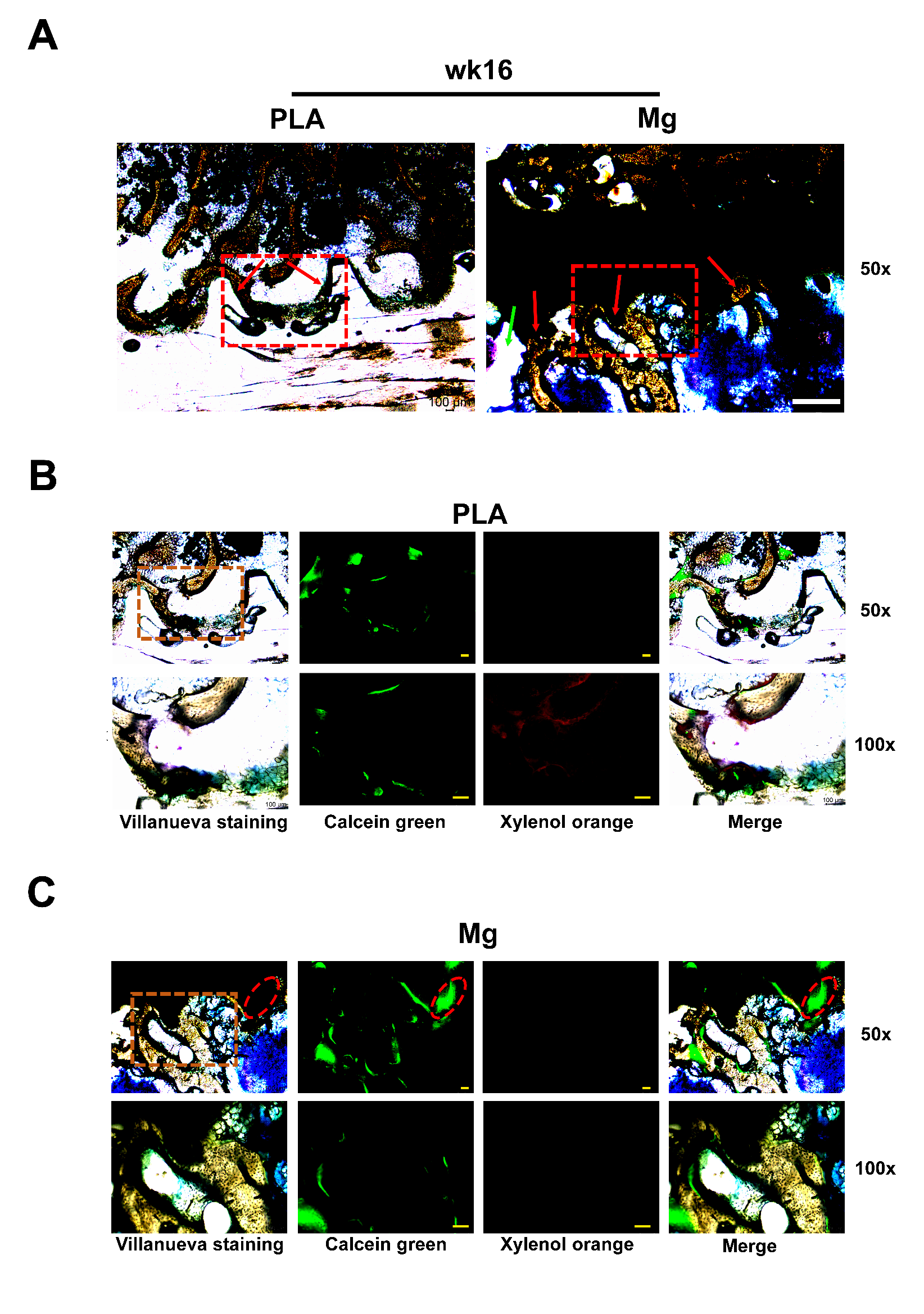


**Supplementary Figure 4.** High-purity Mg screw improved peri-implant bone remodeling at week 16 post-implantation. (A) Representative images of peri-implant tissues stained with Villanueva bone stain at week 16 post-implantation. Red arrows indicated bone-implant contact; green arrows indicated gaps between bone and implant; ROI was marked by a red dashed rectangle; white scale bar = 1 mm. (B and C) Representative images of ROI stained with Villanueva osteochrome bone stain and calcein green/xylenol orange fluorochromes stain at week 16 post-implantation. Regions within the orange dashed box were further magnified; the red dashed circle indicated an acellular calcified matrix layer that was adjacent to the implant surface and positive for calcein green; scale bar = 100 μm.
